# Diagnosis of food allergy and practical management of anaphylaxis: acute management

**DOI:** 10.1186/2045-7022-1-S1-S63

**Published:** 2011-08-12

**Authors:** Graham Roberts

**Affiliations:** 1 Southampton University Hospital NHS Trust, Paediatric Allergy and Respiratory Medicine, University Child Health, Southampton, UK

## 

Anaphylaxis is a paediatric emergency but most cases occur in the community. Children with suspected food allergy need to be assessed and the trigger foods identified (Roberts 2007). The family then require dietetc education to help avoid further exposure (Roberts 2008). With increasing replacement of home-cooked products with manufactured ones, the avoidance of trigger food allergens has become increasingly difficult. It is therefore essential that families, schools, nurseries and other child care professionals understand how to avoid, recognise and manage allergic reactions (Muraro 2007; Muraro 2010). The child and their family therefore need to know how to recognise an allergic reaction and appropriately deal with it. This is facilitated with the use of personalised management plan that take into account the child’s personal risk of anaphylaxis and coexistent medical problems. These plans also need to be shared with the children's other carers. With this approach, we have an opportunity to reduce the future morbidity and mortality that our patients with food allergy experience.
